# Learning environment, stress and coping in psychiatry residents within a national training program: a longitudinal study

**DOI:** 10.1007/s40037-019-0512-7

**Published:** 2019-05-16

**Authors:** Qian Hui Chew, Eric Holmboe, Kang Sim

**Affiliations:** 1 Research Division, Institute of Mental Health, Singapore, Singapore; 2 Milestones Development and Evaluation of the Accreditation Council for Graduate Medical Education, Chicago, Illinois USA; 3 Yale University, New Haven, CT USA; 4 West Region, Institute of Mental Health, Singapore, Singapore

**Keywords:** Learning environment, Psychiatry, Stress, Coping, Residency

## Abstract

**Introduction:**

Perception of one’s learning environment is associated with academic performance and well-being of medical students. Yet, few studies have explored learners’ perceptions and their correlates within a postgraduate environment. This study examined longitudinal changes in learning environment perception, perceived stress and coping for psychiatry residents in junior and senior years of training. Based on extant social learning theories, we hypothesized that learning environment perceptions will improve with seniority, and be accompanied by lower stress levels and better coping.

**Methods:**

Eighty-two out of 101 psychiatry residents (81.2%) from our National Psychiatry Residency Program rated their perception of learning environment, perceived stress, and coping strategies from June 2016 to June 2018. Correlations between these variables, and changes across five timepoints were examined.

**Results:**

Senior-year residents reported better perception of learning environment over time, together with increased use of problem-focused coping and lowered perceived stress levels. Junior-year residents reported no changes in learning environment perception and coping strategies, despite rating greater perceived stress levels over time. Perception of learning environment negatively correlated with perceived stress levels and specific coping strategies.

**Discussion:**

Based on these findings, we suggest specific strategies with the emphasis on context, participation, and social interaction within a community of practice to better support residents in training, which are applicable to other similar training programs.

## What this paper adds

Optimization of the learning environment can potentially lead to better academic performance and well-being of the learner including better quality of life and less psychological distress. However, compared with undergraduate medical education contexts, few studies have longitudinally examined the perceptions of residents and their correlates, such as with perceived stress and coping levels within a postgraduate learning environment. When viewed through the lens of ‘situativity theory’ and ‘community of practice’, our findings allow us to suggest strategies with the focus on context, participation, and social interaction to better support our learners and which are applicable to other training environments.

## Introduction

The learning environment has been defined as ‘the atmosphere experienced by the learner in the school or program, essentially, what is valued, recognized and encouraged’ [1]. It highlights the importance of paying attention not just to the physical, objective environment (e.g. amenities) but also the learner’s subjective perceptions [2] of the people (e.g. peers, faculty, social support) and program (e.g. curriculum, teaching) [3]. Psychiatry residency is one of the major disciplines within postgraduate medical specialty training internationally and is exacting in the breadth and depth of the knowledge covered, extent of clinical postings, and slew of required formative and summative assessments during training. To ensure learner wellness for optimal learning, it is imperative to understand how our psychiatry residents view the learning environment, and its relationship with other learner factors including perceived stress levels and coping methods.

Our recent reviews of the use of the Dundee Ready Educational Environment Measure (DREEM) and Postgraduate Hospital Educational Environment Measure (PHEEM) in the context of undergraduate and postgraduate health professions education respectively revealed several important findings and knowledge gaps [4, 5]. Compared with undergraduate education in the health professions, there is a dearth of studies examining the learning environment and its interrelationships with other correlates within postgraduate education including psychiatry. For example, DREEM scores are inversely associated with academic achievement [6–8], and positively correlated with quality of life [9], resilience levels [9], positive attitudes toward psychiatry [10], mindfulness [11], and perceived preparedness for practice [12] in undergraduate medical students. Poor academic self-perception has also been associated with psychological distress and poor peer support [13]. In contrast, PHEEM scores have only been correlated positively with in training examination scores and negatively with burnout measures within postgraduate training [5]. Moreover, there were inconsistent findings regarding the influence of seniority in training on learners’ perceptions of their learning environment [5]. In particular, whilst four studies reported junior trainees having a more favourable perception of their learning environment [14–17], three studies found that senior trainees reported higher scores instead [18–20]. Greater perceived stress as a result of problems with the learning environment (e.g. duty-hours limits not enforced, academic overload) has been shown to negatively affect sleep quality in psychiatry residents regardless of residency year [21], as well as cortical plasticity in a sample of graduate students [22]. This behoves a closer examination of how the different factors within a learning environment affect outcomes in postgraduate medical education. Additionally, there is a paucity of studies examining longitudinal changes related to learning environment [5].

Germane to understanding the learning environment, the situativity theory suggests that learning is situated in the learner’s experience and occurs within a context with the need to consider areas such as the community of learners, culture, and the external environment [23]. Learning outcomes can therefore differ between learners due to complex interactions among factors that constitute one’s experience [23]. Knowledge can be formed internally through an interaction between one’s experience and the environment [24, 25], or in a social context before adoption by individuals [26] through a process called collaborative elaboration [27, 28]. The formation of such communities of practice gives learners an opportunity to develop personally and professionally [29].

Given the importance of understanding the learning environment and paucity of such literature within postgraduate psychiatry training, our study aimed to explore the learning environment as perceived by residents in junior and senior years of training within a National Psychiatry Residency Program in Singapore, and its inter-relationships with other correlates over time. Based on aforementioned social learning theories, we hypothesized that residents in senior years of training would report better scores on learning environment with accompanied improvements in perceived stress and coping strategies adopted.

## Methods

In 2010, Singapore adopted an Accreditation Council for Graduate Medical Education-International (ACGME-I) accredited residency training program covering multiple specialties (including psychiatry) with pre-determined standards for the learning environment, including specific content coverage within the curriculum, competency milestones and working hours. Within the National Psychiatry Residency Program, 4 out of the 5 years are accredited by ACGME-I and the final year by our local Joint Commission on Specialist Training within Ministry of Health, Singapore.

In this study, psychiatry residents across five residency years within the National Psychiatry Residency Program in Singapore were invited to participate. The same scales were administered to recruited residents every 6 months over a period of 24 months from June 2016 until June 2018, with a total of five timepoints including the baseline. This study was approved by the Institutional Review Board of the National Healthcare Group, Singapore (NHG DSRB Ref: 2015/01139). The study was carried out in accordance with the Declaration of Helsinki, with there being no potential harm to participants, guaranteeing the anonymity of participants, and obtaining the informed consent of all participants.

### Data collection

The following demographic data were included in the data collection form: date of birth (month and year), gender, marital status, and year of residency. We employed scales assessing residents’ perceptions of the learning environment [30], perceived stress levels [31], and coping mechanisms used [32]. To ensure anonymity, these questionnaires were administered by a research assistant who was not involved in the National Psychiatry Residency Program. Individual names were not collected and all participants were only assigned a code number to facilitate tracking of responses across timepoints.

### Rating scales

The PHEEM questionnaire [30] was used to assess residents’ perceptions of their learning environment. The scale consists of 40 items with three subscales, namely Perceptions of Role Autonomy (14 items, maximum score of 56), Perceptions of Teaching (15 items, maximum score of 60), and Perceptions of Social Support (11 items, maximum score of 44). Summation of the three subscale scores provides an overall score ranging from 0 to 160, with higher scores indicating a more favourable perception. The PHEEM is a highly reliable, valid and practical instrument used in many studies conducted in different residency training sites worldwide [5, 15, 33, 34].

The level of stress experienced by the residents in the past month was assessed using the Perceived Stress Scale [31]. The PSS contains 16 items on a four-point Likert scale. The score ranges from 0 to 40, with higher scores indicating a higher level of stress.

The Brief COPE Inventory [32] was used to assess how frequently residents adopted different types of coping strategies. There are 28 items on a four-point Likert scale, and it consists of four domains: active-avoidance coping, problem-focused coping, positive coping, and religious/denial coping [35]. The items are summated for each subscale separately. Higher scores indicate more frequent use of that particular coping strategy.

### Statistical analyses

All analyses were conducted using IBM SPSS 23 (IBM Corp, Armonk, NY [36]). To investigate the variable of residency year, our cohort was split into residents in junior (1st and 2nd years) and senior years (3rd, 4th, and 5th years) of training based on wider scope and depth of clinical work and responsibility, henceforth termed *junior-year* and *senior-year* residents respectively. This was defined by residency year at baseline, or the first time the scales were administered. Paired samples T‑tests for each group were then conducted to examine the point in residency at which there were significant changes as compared with baseline for the various measures, with adjustment for multiple comparisons.

We also conducted Spearman correlations between PHEEM, and Brief COPE and PSS scores for all timepoints in order to examine interrelationships between perceived learning environment and these learner factors (coping strategies, stress levels).

## Results

Overall, 82 out of 101 (81.2%) residents took part in the study at baseline, and 58.5% (*n* = 48) were male. There was an equal proportion of junior-year (50.0%) and senior-year (50.0%) residents within the study. Among junior-year residents, 63.4% (*n* = 26) were single and of the senior-year residents, 48.8% (*n* = 20) were single.

### Comparisons of scale scores within junior and senior residents

Table 1 shows the comparisons of scale scores within junior and senior residents across timepoints. Regarding PHEEM, there were no significant differences between timepoints for total and subscale scores observed for junior-year residents. This was in contrast to senior-year residents whose total PHEEM scores increased between timepoints 2, 3, and 4 as compared with baseline (all *p* ≤ 0.005). The same pattern (improvement at timepoints 2, 3, 4) was observed for all PHEEM subscales over time compared with baseline (all *p* ≤ 0.01).

**Table 1:** Comparison of scale scores (with baseline) for 82 junior and senior psychiatry residents in the National Psychiatry Residency Program (Singapore) from June 2016 to June 2018

Scale	Mean (SD)	*t*	*Df*	*P value*
*Junior*				
Perceived Stress Scale				
TP 1 vs TP 4	24.8 (5.36) vs 28.7 (4.53)	−3.445	24	0.002
*Senior*				
PHEEM total				
TP 1 vs TP 2	108.8 (14.0) vs 120.0 (15.5)	−3.04	26	0.005
TP 1 vs TP 3	107.9 (14.2) vs 122.9 (16.7)	−3.99	24	0.001
TP 1 vs TP 4	107.0 (14.8) vs 122.9 (17.4)	−4.29	21	<0.001
PHEEM RA				
TP 1 vs TP 2	38.4 (0.97) vs 42.0 (1.06)	−3.02	28	0.005
TP 1 vs TP 3	38.2 (5.35) vs 43.0 (5.37)	−3.90	25	0.001
TP 1 vs TP 4	37.8 (5.63) vs 42.6 (6.22)	−4.07	21	0.001
PHEEM SS				
TP 1 vs TP 2	28.4 (4.37) vs 31.6 (4.48)	−2.79	27	0.010
TP 1 vs TP 3	28.1 (4.44) vs 32.9 (5.46)	−3.50	24	0.002
TP 1 vs TP 4	27.7(4.53) vs 33.2 (5.49)	−3.81	21	0.001
PHEEM T				
TP 1 vs TP 2	42.0 (5.77) to 45.9 (5.60)	−2.74	29	0.010
TP 1 vs TP 3	41.8 (6.04) vs 46.8 (6.38)	−3.62	25	0.001
TP 1 vs TP 4	41.4 (6.29) vs 47.4 (6.36)	−4.19	22	<0.001
Perceived Stress Scale				
TP 1 vs TP 3	26.8 (5.08) to 24.2 (5.92)	2.72	24	0.012
TP 1 vs TP 4	27.2 (5.00) vs 24.3 (5.76)	3.29	22	0.003
COPE Problem-focused				
TP 1 vs TP 5	17.9 (3.81) vs 20.0 (4.31)	−3.43	10	0.006

Junior residents defined as residents in Year 1 and 2 of training. Senior residents defined as residents in Years 3, 4, and 5 of training

*COPE* Brief COPE Inventory, *PHEEM* Postgraduate Hospital Educational Environment Measure, *RA* Role Autonomy subscale, *SS* Social Support Subscale, *T* Teaching subscale, *TP* timepoint

For the PSS, junior-year residents experienced an increase in scores indicating greater stress levels at timepoint 4 compared with baseline (*p* = 0.002). Conversely, senior-year residents reported a decrease in stress scores at timepoints 3 (*p* = 0.012) and 4 (*p* = 0.003) compared with baseline.

For the Brief COPE scale, no significant differences were observed for junior-year residents across timepoints compared with baseline. A significant increase in problem-focused coping was noted for senior-year residents at the final timepoint when compared with baseline (*p* = 0.006).

### Correlations between total PHEEM and PSS scores

A significant negative correlation was noted between total PHEEM scores and PSS for both junior (r_s_ = −0.429, *p* < 0.001) and senior residents (r_s_ = −0.347, *p* < 0.001).

### Correlations between total PHEEM and Brief COPE scores

Total PHEEM scores were negatively correlated with active-avoidant coping only for junior-year residents (r_s_ = −0.288, *p* < 0.001), and negatively correlated with religious/denial coping only for senior-year residents (r_s_ = −0.178, *p* < 0.05).

## Discussion

There are several main findings in this longitudinal study. First, whilst junior-year residents had no significant changes in perception of learning environment and coping strategies, there was an increase in the level of perceived stress at timepoint 4 as compared with baseline. Second, for senior-year residents, a decrease in perceived stress level was observed at timepoints 3 and 4 compared with baseline. This was accompanied by better perception of their learning environment (overall, role autonomy, social support, teaching) and adoption of problem-focussed coping over time. Overall perception of learning environment and perceived stress scores were negatively correlated regardless of seniority. Different coping strategies were correlated with overall perception of learning environment when junior and senior resident groups were examined separately.

### Findings related to junior-year residents

For junior-year residents, the perception of their learning environment and coping methods did not appear to change over the timepoints. Additionally, they experienced higher stress levels at timepoint 4 as compared with baseline. These findings can be understood from situativity theory [23] and an understanding of community of practice [29]. New learners (junior-year residents) join a community of practice initially from the edge and learn through a process of legitimate peripheral participation [37]. Situated learning encompasses not only the acquisition of knowledge and concepts, but also all that is inherent within the learning environment, including the social interactions [23] which are measured by PHEEM, and thus may not seem to change much early on in training. Furthermore, junior-year residents may have fewer opportunities to make independent clinical decisions as they are part of an inter-professional team with more experienced colleagues, contributing to a perception of reduced autonomy. Concomitantly, coping strategies can moderate levels of stress experienced during training, and the lack of coping strategy modification (positive and negative) found in junior-year residents over time could have contributed to greater perceived stress at timepoint 4. Poorly managed stress may subsequently influence their perception of the learning environment [38].

### Findings related to senior-year residents

On the other hand, perceptions of their learning environment for senior-year residents improved over time. Additionally, they experienced lower stress levels, and had increased use of problem-focused coping. These observations could be explained by their progress into full participation within communities of practice [29] and involvement in the process of collaborative elaboration [27, 28]. Senior-year residents are more likely to have had opportunities to establish working relationships with fellow residents during various clinical rotations. These shared experiences provide a basis for them to reflect on their own perspectives in a community setting, which generates knowledge that can be shared amongst learners [27, 28]. This enhances both personal and professional development [29] and could contribute to the perception of a learning environment that is supportive and intellectually stimulating. Senior-year residents were also noted to have significantly increased their use of problem-focused coping strategy later in their training. This could indicate attempts at adjusting coping strategies which possibly attenuated the effect of accumulated stress throughout residency. Lower stress levels in turn contributed to more positive perceptions of their learning environment [38].

### Correlations among learning environment, stress levels, and coping strategies

Stress levels were significantly correlated to various domains of perception of learning environment for both groups of residents in junior and senior years of training across multiple timepoints, reinforcing findings by Llera and Durante [38]. This underscores the need to take into consideration the bidirectional influence between learning environment and stress. Educators should consider the domains of role autonomy, social support, and teaching as critical avenues of early intervention for residents.

Different coping styles were associated with better perception of learning environment in junior and senior residents, with less active-avoidant coping for junior-year residents and less religious/denial coping for senior-year residents reaching significance. Coupled with the finding that coping styles of junior-year residents did not change significantly over time, poor perceptions of learning environment could be resistant to change in junior-year residents who prefer active-avoidant coping. As various learning outcomes [6–8] and the well-being of residents [9] could be negatively affected by poor perceptions of their learning environment, educators may consider prioritizing the promotion of functional coping strategies in residents.

### Ways to help residents in junior and senior years of training

How then can junior and senior psychiatry residents be further assisted in their learning journey? The basic tenets of situativity theory [23] and community of practice [29] are that learning takes place within a context and through social interactions respectively. For junior-year residents, the frame of interconnecting components of learning (identity, meaning, practice, community) within a community of practice can be considered [29]. These four components are associated with ideas about learning as becoming, experience, doing and belonging respectively. First, for learning as becoming, socialization of these residents into the specialty is useful for creating a supportive learning environment. This can be done by pairing them with senior buddies who can encourage the development of positive coping strategies and give advice about professional training, which can aid the development of professional identity. An undergraduate study involving first-year medical students has also shown that one main contributor to perceived stress is difficulty in establishing contact with other medical students [39]. Second, for learning as experience, closer supervision especially at the incipient stage of training can be crucial in guiding professional development. Third, for learning as doing, active involvement of junior-year residents in clinical decisions and case management within inter-professional teams can also move them from legitimate peripheral participation [37] to full participation over time, enhancing perceived role autonomy. Fourth, for learning as belonging, inclusion of residents in junior years within professional activities such as continuing medical education sessions and non-academic activities can foster a greater sense of being part of the specialty to which they have committed their time and effort.

Pertaining to senior-year residents, three essential constituents of a community of practice [29], namely mutual engagement, joint enterprise and shared repertoire, can be a useful frame to further aid their learning within the learning environment [40]. Mutual engagement refers to interaction and participation in work and non-work related activities [40]. In this regard, senior-year residents can be involved as both a mentor, through the aforementioned buddy system, and a mentee, by being assigned a mentor for personal guidance. This increases opportunities for exchange of ideas and reflective learning with colleagues of varying levels of experience during training. Joint enterprise refers to the need for the group to respond to an internally created mandate rather than an external one [40]. A sense of role autonomy and positive perception of the learning environment [30] can be fostered by allowing senior-year residents to take charge of some portion of their training (e.g. curriculum planning together with faculty). Shared repertoire refers to the routines, tools, and ways of doing things that the community has adopted [40]. In this context, senior-year residents can be incrementally involved in training juniors, which can help develop a shared purpose and processes in clinical practice. The PHEEM rating scale [30], which captures domains of perceptions of role autonomy, teaching and social support, can potentially be administered over time to ascertain the changes within the learners at different levels of seniority in the perception of their learning environment following the implementation of these suggested measures.

There are several limitations of the study. First, despite being a National Psychiatry Residency Program, the overall intake per year is still small compared with other similar Psychiatry Residency Programs internationally. Second, despite our best efforts, there is greater attrition at the last timepoint of follow-up. Third, inclusion of other factors such as personality variables and contemporaneous life events within reasonable test burden could have proffered further insights into other contributors towards the resident’s perceived learning environment.

In conclusion, we found differences between junior and senior psychiatry residents in that the latter had better perceptions of their learning environment over time related to role autonomy, teaching and social support with a corresponding reduced level of perceived stress and increased employment of problem-focused coping. This is consistent with theories of learning emphasizing the importance of context, participation, and social interaction within a community of practice to facilitate learning. Based on these findings and associated theories of learning, we have suggested several ways to further enhance learning for these psychiatry residents within a National Residency Program, which are applicable to other similar training programs.
